# An Unusual Simultaneous Occurrence of Diabetes and Hypothyroidism in a Periodontitis Patient: A Case Report

**DOI:** 10.7759/cureus.33844

**Published:** 2023-01-16

**Authors:** Ajay M Khade, Jyoti Khade, Rashmi Nagdeve, Mangesh Phadnaik, Obaid Noman

**Affiliations:** 1 Department of Pharmacology, Datta Meghe Medical College, Datta Meghe Institute of Higher Education and Research, Nagpur, IND; 2 Department of Periodontology, Government Dental College and Hospital, Nagpur, IND; 3 Department of Medicine, Government Medical College and Hospital, Nagpur, IND; 4 Department of Pathology, Jawaharlal Nehru Medical College, Datta Meghe Institute of Higher Education and Research, Wardha, IND

**Keywords:** plaque, calculus, hypothyroidism, periodontitis, diabetes mellitus

## Abstract

Periodontal disease, diabetes mellitus, and hypothyroidism are commonly prevalent non-communicable diseases afflicting the human population all over the world, with the added burden on the health care system increasing the overall morbidity. It has been seen through various shreds of evidence that systemic diseases may influence the course of the localized disease and vice versa.

Here, we report a case of 38-year-old female periodontitis patient also diagnosed with diabetes mellitus and hypothyroidism. Periodontitis is one of the complications of diabetes. But the occurrence of periodontal disease is a less common intra-oral finding in hypothyroidism as compared to diabetes. All these three chronic diseases were simultaneously observed in this patient, which is a rare occurrence and can adversely affect her overall prognosis. This case report highlights the need for a systematic interdisciplinary approach in the diagnosis and management of such cases.

## Introduction

Periodontal diseases are a complex group of diseases with a multifactorial origin that involves a complex interaction between the host, the subgingival microbiota, and environmental modifying factors destroying the tooth-supporting structures [1]. Various systemic diseases can show manifestations on the gums (gingiva and tooth-supporting structures), with variable nature and severity. These include diseases like diabetes mellitus, congenital disorders, Crohn's disease, other primary immunodeficiencies, and some conditions that affect mainly the gingiva causing enlargement, ulceration, red and white lesions, and hyperpigmentation. Some rare systemic conditions also aggravate pre-existing periodontitis such as Papillon-Lefèvre syndrome and leucocyte adhesion deficiency [2].

Diabetes mellitus, which is a commonly prevalent disease, shows a wide variety of effects on periodontal health. Diabetes acts as a crucial modifier in periodontal diseases and it is a fact that periodontitis is considered the sixth complication of diabetes [3]. The glycemic control levels in diabetes help in deciding the grading of periodontitis. Strong evidence exists about mechanisms and pathways in periodontitis pathogenesis in patients with diabetes [3]. Few studies have demonstrated that periodontal dysbiosis/disease leads to the development of insulin resistance and may aggravate pancreatic β-cell failure. Treatment of periodontal disease leads to a reduction in glycated hemoglobin in diabetic patients. Oxidative stress may be another vital link between diabetes and periodontitis as it can activate proinflammatory pathways common to these diseases [4].

Similarly, thyroid dysfunction, i.e., hypothyroidism or hyperthyroidism, is another commonly occurring disorder of the endocrine system. Thyroid dysfunction causes an imbalance of homeostasis in the body and adversely affects tissue repair. Growth regulation, development of the body, and its metabolic functions are maintained normally by the thyroid hormones [5,6]. Dysgeusia, macroglossia, changed tooth morphology, slow wound healing, and slow tooth eruption rate are characteristic oral findings in hypothyroidism [7]. Low circulating free T4 levels and elevated thyroid stimulating hormone (TSH) levels >5.5 μIU/ml are suggestive of hypothyroidism [8]. It is a very common disease in adult Indian individuals, especially females.

Systemic disorders and conditions have been shown to alter host response and physiology impairing immune defense against periodontal pathogens and host barrier function. This creates opportunities for periodontal destruction. These disorders and conditions themselves do not cause periodontal disease, but they may predispose, accelerate or increase its progression. Here, we report a case of periodontitis in a female who was concurrently diagnosed with both diabetes and hypothyroidism.

## Case presentation

**Figure 1:**
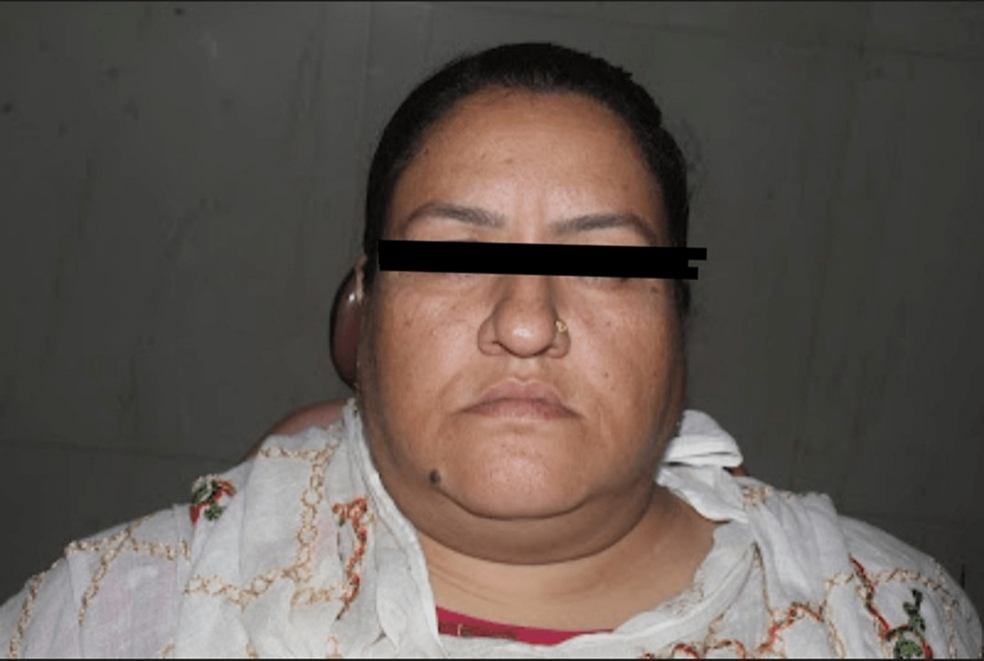
Extraoral view of the patient showing puffiness of the face

**Figure 2:**
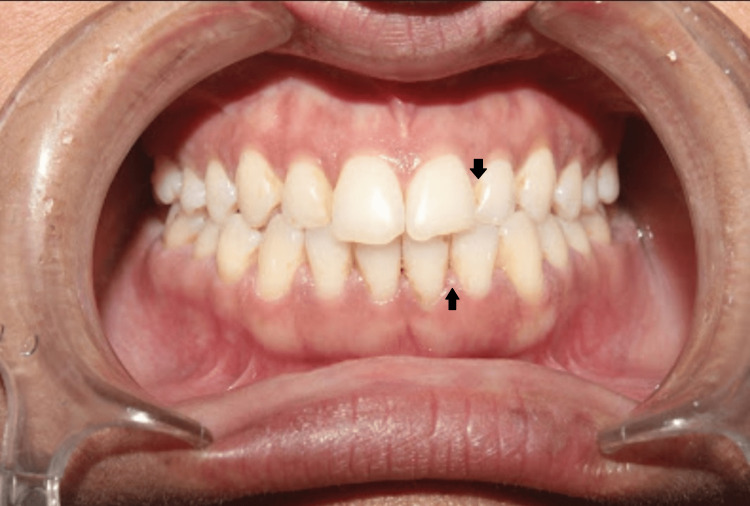
Intraoral clinical photograph showing interdental deposits (black arrows) in both arches

**Figure 3:**
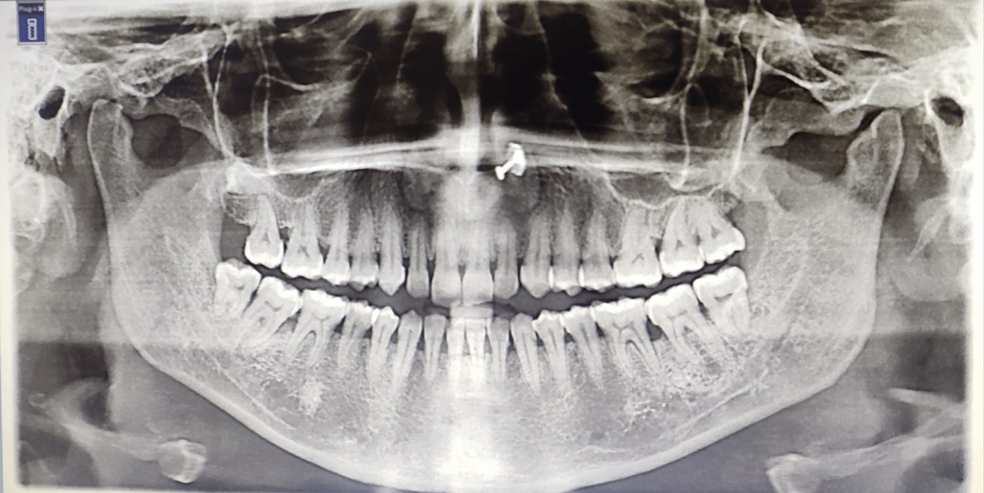
Orthopantomogram showing a generalized horizontal bone loss with localized areas of vertical bone loss

**Figure 4:**
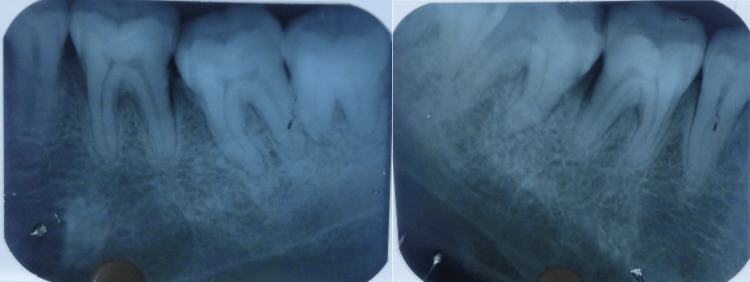
Intraoral periapical radiographs of the mandibular premolar/molar region showing a generalized horizontal bone loss and vertical bone loss around first molars

A 38-year-old female patient came to the periodontology department with complaints of deposits on teeth present for two months and bleeding gums for two weeks. The patient was apparently alright six months back when she was diagnosed with hypothyroidism. The physician prescribed a tablet of thyroxine sodium 50 mcg/day. She was also recently diagnosed with diabetes mellitus just two weeks before reporting to the periodontology clinic. The physician prescribed tablet metformin 500 mg once daily. She complained of weight gain, puffiness over her face and fatigue (Figure 1). Her complete blood count was within normal limits except for total leukocyte count (TLC) and platelet count, which were raised (Table 1). Her fasting and post-meal blood glucose levels were also raised. The thyroid function test showed elevated TSH levels (Table 2). An intraoral examination revealed the presence of supra- and subgingival plaque and calculus (Figure 2). Many teeth showed clinical attachment loss of 2-3 mm with deeper pockets (4-5 mm) and attachment loss (4-5 mm) in mandibular molars of both sides. Bleeding on probing was present in more than half of the evaluated teeth sites. Radiological examination revealed generalized horizontal bone loss with vertical bone loss in molars (Figures 3, 4).

**Table 1:** Haematological investigations

Investigations	Result	Normal range
Hemoglobin	14 gm/dl	12-15 gm/dl
Red blood cell count	4.78 million/cubic mm	3.8-4.8 million/cubic mm
Packed cell volume	40%	40%-50%
Mean corpuscular volume	83.2 fl	83-101 fl
Mean corpuscular hemoglobin	29.4 pg	27-32 pg
Mean corpuscular hemoglobin concentration	35.3%	31.5%-34.5%
Red cell distribution width - coefficient of variation	13%	11.6%-14.0%
Total leukocyte count	12,100 cell/cubic mm	4000-10,000 cell/cubic mm
Platelet count	420,000/cubic mm	150,000-410,000 /cubic mm
Fasting blood glucose	117 mg/dl	70-100 mg/dl
Post-meal blood glucose	219 mg/dl	Up to 160 mg/dl

**Table 2:** Thyroid function test T3, triiodothyronine; T4, tetraiodothyronine; TSH, thyroid stimulating hormone

Investigations	Results	Normal range
Serum T3	1 ng/ml	0.8-2.0 ng/ml
Serum T4	8.20 ug/dl	5.10-14.10 ug/dl
TSH	4.48 mIU/ml	0.27-4.20 mIU/ml

Based on the clinical examination, history taking, and various investigations, the patient was diagnosed as generalized, stage II periodontitis with grade B disease modified by diabetes and hypothyroidism. The patient was educated about the harmful effects of poor oral hygiene and the intraoral effects of diabetes and hypothyroidism. She was motivated and trained in the correct oral hygiene practices. Under systemic antibiotic coverage with tablet amoxycillin 500 mg thrice daily for one week, she underwent nonsurgical periodontal therapy involving scaling and root planing in multiple appointments. She was also prescribed topical antiseptic gels for gingival massage for about two weeks. She is now kept on recall evaluation and supportive periodontal treatment every two weeks for the next six months, followed by monthly revisits thereafter. Information about her oral condition was given to her treating physician and his consultation was sought prior to oral interventions. Her dental treatment appointments are planned with the physician’s consent and under his supervision. She is kept on a fitness program involving regular exercises, a balanced diet specific to her systemic conditions, and stress management through yoga and meditation.

## Discussion

Periodontitis and non-insulin-dependent diabetes mellitus (NIDDM), i.e., type 2 diabetes, are two non-communicable diseases that show a close association. They are highly prevalent all over the world and compromise the quality of life in the affected individuals. People suffering from type 2 diabetes have double the risk of periodontal disease compared to non-diabetics. The available evidence thus suggests that patients with type 2 diabetes usually have periodontitis simultaneously. Diabetic patients are also more vulnerable to developing severe periodontitis, with an increased risk of poor glycemic control [4].

Intraoral periodontal findings in the present case are consistent with the established shreds of evidence about the relationship between diabetes and periodontal health. Bacteria and their degradation products in periodontal disease may penetrate the host tissues and reach systemic circulation. This can activate an aggravated systemic inflammatory response. Proinflammatory mediator levels are elevated systemically that further facilitates insulin resistance. Advanced glycation end product (AGE) accumulation in diabetic patients may induce an exaggerated monocytic inflammatory response leading to severe periodontitis. Interleukin-1 (IL-1), tumor necrosis factor-α (TNF-α), IL-6, platelet-derived growth factor, and insulin-like growth factor-1 may be produced after AGEs bind to monocyte receptors. This further regulates the transcription of C-reactive protein (CRP), a human acute phase reactant. These cytokines are believed to activate resident cells in periodontal tissues to produce metalloproteinases that cause the destruction of connective tissue including alveolar bone destruction due to osteoclast activation [5,6].

In hypothyroidism, reduced levels of thyroid hormone are available to the target tissues. Serum TSH concentration is considered the most reliable marker of thyroid function. American Thyroid Association guidelines suggest the estimation of serum TSH concentration in every individual at 35 years of age and re-evaluation every five years [9]. This may also be beneficial for determining the role of thyroid hormone imbalance in periodontitis, thereby preventing morbidity related to this condition. Hypothyroid patients show a reduced bone turnover rate that may be secondary to delayed resorption during bone remodeling leading to bone mass increase. It has also been observed that in hypothyroidism patients, there is delayed wound healing that occurs as a result of decreased metabolic activity of the fibroblasts. Delayed wound healing can further increase the risk of infections [10,11]. TNF-α and IL-6 are also increased in hypothyroidism, which play an important role in osteoclast differentiation and function. These cytokines enter the periodontium through systemic circulation and may stimulate the resident cells to produce matrix metalloproteinases that cause connective tissue destruction and alveolar bone loss by activating osteoclasts [12-14]. On the other hand, periodontitis itself can act as a chronic reservoir of microparticles released by inflammatory cells that may have a role in the pathophysiologic process of various systemic diseases [15].

The patient in the present case reported bleeding gums and showed radiological evidence of bone loss with moderate amounts of local factors, which can be attributed to her susceptibility to infection owing to pre-existent diabetes and hypothyroidism. The combined systemic influence of these conditions may be the reason behind an exaggerated response to the plaque microbes. This might be one of the first reported cases presenting with periodontitis, diabetes, and hypothyroidism simultaneously.

## Conclusions

Periodontists should evaluate periodontal disease patients for the presence of diabetes and thyroid dysfunction, and similarly, endocrinologists should investigate for the presence of periodontitis in diabetic and/or hypothyroidism patients, as early diagnosis and prompt interventions in these conditions will be hugely beneficial for patients’ overall well-being.
